# Weak whole‐plant trait coordination in a seasonally dry South American stressful environment

**DOI:** 10.1002/ece3.3547

**Published:** 2017-11-09

**Authors:** José L. A. Silva, Alexandre F. Souza, Adriano Caliman, Eduardo L. Voigt, Juliana E. Lichston

**Affiliations:** ^1^ Programa de Pós‐Graduação em Ecologia Universidade Federal do Rio Grande do Norte (UFRN) Natal Brazil; ^2^ Departamento de Ecologia UFRN Natal Brazil; ^3^ Departamento de Biologia Celular e Genética UFRN Natal Brazil; ^4^ Departamento de Botânica e Zoologia UFRN Natal Brazil

**Keywords:** Brazil, CSR triangle, leaf biochemistry, leaf‐shedding behavior, litterfall production, plant anatomy

## Abstract

A core question involving both plant physiology and community ecology is whether traits from different organs are coordinated across species, beyond pairwise trait correlations. The strength of within‐community trait coordination has been hypothesized to increase along gradients of environmental harshness, due to the cost of adopting ecological strategies out of the viable niche space supported by the abiotic conditions. We evaluated the strength of trait relationship and coordination in a stressful environment using 21 leaf and stem traits of 21 deciduous and evergreen woody species from a heath vegetation growing on coastal sandy plain in northeastern South America. The study region faces marked dry season, high soil salinity and acidity, and poor nutritional conditions. Results from multiple factor analyses supported two weak and independent axes of trait coordination, which accounted for 25%–29% of the trait variance using phylogenetically independent contrasts. Trait correlations on the multiple factor analyses main axis fit well with the global plant economic spectrum, with species investing in small leaves and dense stems as opposed to species with softer stems and large leaves. The species’ positions on the main functional axis corresponded to the competitor‐stress‐tolerant side of Grime's CSR triangle of plant strategies. The weak degree of trait coordination displayed by the heath vegetation species contradicted our expectation of high trait coordination in stressful environmental habitats. The distinct biogeographic origins of the species occurring in the study region and the prevalence of a regional environmental filter coupled with local homogeneous conditions could account for prevalence of trait independence we observed.

## 1 INTRODUCTION

Variation in morphology, physiology, and phenology of leaves, stems, and roots allows plant species to take up different amounts of water and nutrients along temporal and spatial gradients of soil resources and light availability. Variation in plant form and function creates the basis for species coexistence, plasticity, and evolvability (Kleyer & Minden, 2015). A core question involving both plant physiology and community ecology is whether multiple traits of different organs covary across co‐occurring species (hereafter, trait coordination), beyond pairwise correlations between traits (Kleyer & Minden, 2015; Reich, 2014; Reich et al., 2003). Most research carried out in the last decade has focused on integrative macro‐morphological traits such as plant height, leaf area, and wood density (e.g., Diaz et al., 2016; Westoby, Falster, Moles, Vesk, & Wright, 2002; Wright, Falster, Pickup, & Westoby, 2006). Recently, functional ecology has embraced anatomical and biochemical traits, which are known to play important adaptive roles (Li et al., 2015; Somavilla, Kolb, & Rossatto, 2014). For example, the palisade parenchyma layer has been positively correlated with specific leaf area (the ratio of blade area to leaf dry mass content, SLA), which is involved in the leaf's potential for photosynthesis (Markesteijn, Poorter, & Bongers, 2007). In addition, the palisade parenchyma concentrates the largest fraction of leaf nitrogen and ultimately indicates the leaf's potential for the synthesis of biosynthetic precursors and cellular fuels (Li et al., 2015; Reich et al., 2003; Somavilla et al., 2014). In stems, the negative correlation between xylem vessel diameter and wood density influences the resistance to drought‐induced cavitation and the capacity for water storage (Chave et al., 2009).

The notion that traits coordinate along axes of variation has been used to represent the trade‐offs underlying ecological strategies (Chave et al., 2009; Wright et al., 2006). Functional variation is central to theories of plant strategy such as that of Grime's (1997) CSR triangle, which has found growing application in distinct ecosystems (Pierce et al., 2016). The CSR framework defines a triangular trait space between extreme strategies that maximize resource acquisition in productive environments (competitive strategy), survival in stressful conditions (stress‐tolerant strategy), and short lifespan where disturbances are frequent (ruderal strategy). The transition from the competitive to the stress‐tolerant strategy in the CSR triangle finds correspondence in the “fast‐slow” economic spectrum of leaves, which runs from resource‐acquisitive leaves with high specific leaf area, high nitrogen and phosphorous content, fast leaf gas exchange, but short lifespan, to resource‐conservative leaves with an opposite set of traits (Wright et al., 2004). Accordingly, the wood economic spectrum ranges from low‐density stems with tissues that facilitate growth and water movement but have reduced resistance to embolism and physical damages, to slow growth stems with an opposite set of traits (Chave et al., 2009). The correlation between SLA and deciduousness (Méndez‐Alonzo, Paz, Cruz, Rosell, & Olson, 2016) as well as between SLA and ecosystem productivity (Reich, 2014) suggests that litterfall production is correlated to the leaf economic spectrum. Deciduous and evergreen leaves occur at opposite ends of this spectrum (Fu et al., 2012; Méndez‐Alonzo et al., 2016).

The strength of within‐community trait coordination has been hypothesized to increase along gradients of environmental harshness while niche space decreases, due to the cost of adopting ecological strategies out of the viable niche space supported by the abiotic conditions (Dwyer & Laughlin, 2017a,b; Westoby & Wright, 2006). Under this hypothesis, environmental harshness variation would drive a gradient of communities presenting loose to strongly constrained niche spaces and, thereby, trait coordination. Evidence supporting many viable combinations of leaf and stem traits within a loose niche space has been found in natural systems such as nonseasonal tropical regions (Baraloto et al., 2010; Fortunel, Fine, & Baraloto, 2012). On the other hand, stronger coordination linking leaf and stem traits within a more constrained niche space has been found in seasonally dry regions (Ishida et al., 2008; Markesteijn, Poorter, Bongers, Paz, & Sack, 2011; Méndez‐Alonzo et al., 2016; Vinya, Malhi, Brown, & Fisher, 2012). Here, we evaluated the strength of trait relationship and coordination in a heath vegetation community growing on a seasonally dry and soil‐poor coastal plain in northeastern South America. We used 21 leaf and stem traits of 21 deciduous and evergreen woody species, including traits that are missing in the whole‐plant economic context, such as anatomical and biochemical traits, and litterfall production.

The *Restinga* heath vegetation is a mosaic of herbaceous, open scrub, and short dense forests that cover coastal sandy plains of eastern South American, mostly along the Brazilian coast. Heath vegetation communities are recent, an only colonized plains produced by marine transgression in the last 7,000 years (Scarano, 2002). They have few endemics species, and most constituent species also occur in neighboring species‐rich ecosystems such as the semi‐arid Caatinga, Cerrado savanna, and Atlantic and Amazon rainforests (Scarano, 2002). Colonizing species have reduced productivity (Pires, De Britez, Martel, & Pagano, 2006) and display physiological ability to deal with harsh conditions that include seasonal drought, heat stress, and sandy soils with poor nutrients and water retention (Brancalion, Vidal, Lavorenti, Batista, & Rodrigues, 2012). We expected the stressful seasonal drought (6–9 months long) and nutrient‐poor soils to produce highly coordinated traits.

## 2 MATERIAL AND METHODS

### 2.1 Study area

Data were collected at the Barreira do Inferno Launch Center, Rio Grande do Norte state, northeastern Brazil; see Silva, Souza, Jardim, and Goto (2015) for further details on the study area and a description of the plant community composition. The Launch Center is a 1900 ha coastal area containing tall (ca. 80 m a.s.l.) sandy dunes along the sea line and sandy plains (ca. 40 m a.s.l.) punctuated by short palaeodunes (Muehe, Lima, & Lins‐de‐Barros, 2006). The climate is tropical with a severe dry summer (Aw Köppen climate type, Alvares, Stape, Sentelhas, De Moraes Gonçalves, & Sparovek, 2013). Mean annual temperature is 26°C and mean annual precipitation is 1,746 mm (INMET 2014). Myrtaceae is the most abundant botanical family in the study area.

### 2.2 Data collection

We examined 14 leaf and stem traits of the 33 most abundant species in the study area (Table S1). For 21 of these species, seven additional traits related to leaf anatomy and biochemistry were measured. Leaf and stem samplings were collected from 80 25‐m² plots distributed along 16 transects of 100 m long (five plots per transect). Whenever possible, we collected the organs from the same individuals. Leaves were collected within a 2‐month interval to reduce temporal variation in leaf biochemistry. To encompass as much phenotypic variability as possible, we sampled plants that were a minimum of 5 m apart, although they were often hundreds of meters to a few kilometers apart.

Leaf dry mass, leaf moisture, leaf area, and SLA were determined for five fully expanded sun leaves with little to no damage from 10 mature individuals per species (Pérez‐Harguindeguy et al., 2013). We calculated leaf blade area using the ImageJ program (https://imagej.nih.gov/ij/) from the images of scanned leaves. Three leaves per individual per species were fixed in 70% (v/v) ethanol until anatomical analysis was performed. Freehand transections of each leaf blade were obtained and stained with alcian blue and safranine. Leaf thickness, mesophyll layer, palisade and spongy parenchyma, and cuticle were measured (in μm) using a Nikon Eclipse E200 microscope. We also measured the content of starch and nonreducing soluble sugars (predominantly sucrose) in 200 mg of fresh leaves for each individual per species using the Antrona method (McCready, Guggolz, Siliviera, & Owens, 1950; Van Handel, 1968). The total soluble protein content was estimated by the Bradford's method (Bradford, 1976).

Litterfall production and temporal variability were measured in 45 plots as part of a long‐term plant phenology project. Six 0.125 m² plastic basins were established as litterfall traps in each plot and used for monthly collection from December 2015 to April 2016. The leaf litterfall found in each basin was oven‐dried at 70°C for 72 hr and then sorted by species. Litterfall production has been used as a proxy for primary production of aerial biomass (Clark et al., 2001) and was estimated by dividing the monthly dried leaf mass per species per plot by respective species abundance in the same plot reported in Silva et al. (2015). We considered the temporal variability of litterfall production as an ecological variable related to phenological strategies on how species respond to water stress, and it was measured as the coefficient of variation of species‐specific litterfall production per plot for all 5 months. We evaluated whether the employed method adequately represented the litterfall production for both abundant and rare *Restinga* species by analyzing the correlation between these two variables (temporal variability and mean litterfall production) with the number of litterfall traps in which species were found. Most abundant species (whose litterfall production was captured by a higher number of traps) had similar mean and coefficient of variation values as rare species (captured by a smaller number of litter traps), as suggested by the low coefficients of correlation (Fig. S1). To better distinguish the leaf‐shedding behavior of species, we visually classified them as evergreen, semideciduous, or deciduous.

Stem traits were measured in ca. 15‐cm‐long and 2‐cm‐diameter stem sections collected from five individuals per species at the first lateral bifurcation of each plant (Pérez‐Harguindeguy et al., 2013). Stem density was obtained by dividing the fresh mass by its volume, and stem moisture was obtained by the difference between the fresh and oven‐dried mass (70°C for 72 hr). Three of these dried stems were divided into two parts. One part was used to evaluate the bark's relative dry mass and the other part to measure vessel anatomical traits. Stems were polished with six sandpapers of different texture grades until the anatomical xylem structures were exposed. We then took photographs using a 3.0× Nikon magnifying glass and processed them using the ImageJ program. The equivalent circle diameter of 100 or more xylem vessels from images of each stem was measured (Scholz, Klepsch, Karimi, & Jansen, 2013). Vessel density was quantified as the number of vessels per mm². A stem vulnerability index was calculated using vessel diameter and vessel density (Scholz et al., 2013). The main stem length and stem diameter at soil level were obtained from Silva, Silva, and Souza (2016).

### 2.3 Statistical analyses

We first calculated the mean trait values for each species. As some analyses require a full matrix of data, we estimated unobserved trait values (3% of the data) using multiple imputation with chained equations (MICE) through predictive mean matching with the “mice” function from the mice package (Van Buuren & Groothuis‐Oudshoorn, 2011) in R version 3.1.2 (R Core Team, 2013). MICE procedure operates under the assumption that missing data depend only on observed values and not on unobserved values (missing at random data), and it has been reliably considered in ordination analyses (Dray & Josse, 2015). All trait distributions were significantly skewed and required log‐transformation before further analyses.

Pairwise Pearson correlations were used to test for cross‐species relationships among all 21 traits. *p*‐Values were adjusted for multiple comparisons using the Benjamini–Hochberg procedure (Waite & Campbell, 2006). Independence between species was assumed by conventional statistical methods to examine trait correlations and functional trade‐offs. To avoid the problem of species nonindependence, we used the Felsenstein (1985)'s method of phylogenetically independent contrasts (PIC) with the “apply” function from the ape package (Paradis, Claude, & Strimmer, 2004). A phylogenetic tree was produced using Phylomatic 3 (http://phylodiversity.net/phylomatic/) and the megatree R20120829. Branch lengths were assigned to the initial megatree using the “bladj” function in Phylocom 4.2, with angiosperm nodes aged according to Wikstrom, Savolainen, and Chase (2001). Single nodes were excluded with the “collapse.singles” function from the ape package (Fig. S2).

Coordination between groups of leaf and stem traits was evaluated by multiple factor analyses (MFA, Pages, 2004) using standardized raw data and PIC values with the “MFA” function of the FactoMineR package (Lê, Josse, & Husson, 2008). PICs values were used to confirm the trait coordination after controlling phylogenetic nonindependence. MFA performs a principal component analysis (PCA) for the group of leaf traits and another PCA for the group of stem traits, separately, and then normalizes all its elements using the square root of the first eigenvalues (Pages, 2004). This creates groups of comparable traits by controlling within‐group covariance. Finally, the normalized datasets were merged to form a single matrix, and then, a global PCA was performed. Only significant axes with eigenvalues >1 were retained.

Species were fitted in the CSR plant strategy triangle according to Pierce, Brusa, Vagge, and Cerabolini (2013) using leaf dry mass, SLA, and leaf area. This method involves three steps: (1) a principal components analysis (PCA) of three key leaf traits (leaf area, leaf dry mass content, and leaf specific area), (2) a regression of trait values against PCA axes, and (3) using these regression equations to produce ternary coordinates, which summarizes the PCA dimensions. These dimensions were divided by 100 to determine the proportional contributions of leaf area, leaf dry mass content, and specific leaf area for each species (Pierce et al., 2013, 2016).

## 3 RESULTS

Trait values differed across species from one (palisade to spongy ratio, bark dry mass, stem vulnerability index, vessel density) to two orders of magnitude (leaf dry mass, leaf area) (Tables S2 and S3; Figs. S3 and S4). Removing the phylogenetic bias in pairwise trait comparisons using PIC values produced a higher number of significant correlations relative to the raw dataset (Table 1; Fig. S5).

**Table 1:** Pairwise Pearson correlations between leaf and stem traits, based on raw data (below the diagonal) and phylogenetically independent contrasts (shaded cells above the diagonal)

	Leaf morphology				Productivity		Leaf anatomy				Leaf biochemistry			Stem morphology					Stem anatomy		
	Ldmass	Lmois	Larea	SLA	Litter	Vlitter	Meso	P/S	M/T	Cuticle	Starch	Sucrose	TSP	Smois	Sdens	Bark	Slength	Sdiam	Vdiam	Vdens	Vindex
Ldmass		0.39	**0.97**	**−**0.23	0.18	**−**0.31	**−**0.14	**−**0.04	0.10	0.36	**−**0.32	**−**0.47	**−**0.46	0.47	**−**0.46	0.69	0.11	0.16	**0.54**	**−0.52**	0.47
Lmois	0.30		0.44	0.20	0.46	**−**0.14	0.44	**−**0.11	**−**0.11	**−**0.04	**−**0.03	**−**0.22	0.44	**−**0.01	0.01	0.19	0.12	0.36	0.15	**−**0.31	0.00
Larea	**0.96**	0.29		**−**0.05	0.22	**−**0.28	**−**0.18	**−**0.09	0.20	0.36	**−**0.16	**−**0.50	**−**0.47	0.37	**−**0.35	**0.59**	0.25	0.31	**0.50**	**−0.51**	0.46
SLA	**−**0.07	0.04	0.14		**−**0.03	0.17	0.09	**−**0.38	0.39	**−**0.05	0.45	**−**0.09	**−**0.03	**−**0.18	0.31	**−**0.36	0.24	0.28	**−**0.22	0.17	**−**0.28
Litter	0.20	0.08	0.17	**−**0.02		**−**0.04	0.36	**−**0.10	0.53	**−**0.19	**−0.65**	**−**0.49	**−**0.21	**−**0.10	0.15	0.04	0.40	0.45	0.04	**−**0.07	0.06
Vlitter	**−**0.05	**−**0.23	0.02	0.04	**−**0.21		**−**0.40	0.21	**−**0.30	**−**0.22	0.26	**0.66**	0.04	**−**0.42	0.43	**−**0.45	**−**0.17	**−**0.15	**−0.51**	0.67	**−**0.45
Meso	**−**0.17	0.30	**−**0.46	**−**0.50	0.02	**−**0.29		**−**0.77	**0.74**	0.18	**−**0.01	**−**0.28	**0.64**	0.43	**−**0.42	**−**0.11	0.10	**0.47**	**−**0.15	**−**0.55	**−**0.14
P/S	**−**0.04	0.14	0.04	0.04	0.13	0.16	**−0.61**		**−**0.79	**−**0.37	**−**0.29	0.13	**−0.63**	**−0.63**	0.55	**−**0.02	0.00	**−**0.54	0.00	0.81	**−**0.13
M/T	**−**0.22	**−**0.42	**−**0.17	**−**0.10	0.13	0.06	0.44	**−**0.41		0.11	**−**0.08	**−**0.31	0.32	0.23	**−**0.15	**−**0.18	0.44	0.65	**−**0.26	**−**0.55	**−**0.31
Cuticle	0.26	**−**0.04	0.09	**−**0.27	**−**0.27	0.12	0.39	**−**0.44	0.13		0.15	**−**0.34	0.17	0.01	**−**0.10	**−**0.24	**−**0.22	**−**0.30	0.12	**−**0.44	**0.64**
Starch	**−**0.13	0.10	0.15	0.55	**−**0.36	**−**0.30	**−**0.18	**−**0.22	**−**0.26	**−**0.11		0.24	0.18	0.58	**−**0.54	0.06	**−**0.50	**−**0.21	**−**0.03	**−**0.27	0.38
Sucrose	0.10	**−**0.16	0.08	**−**0.14	**−**0.08	0.21	**−**0.08	0.03	0.05	0.36	**−**0.23		0.20	0.35	**−**0.34	**0.64**	**−**0.48	0.11	0.29	0.22	0.03
TSP	**−**0.18	0.09	**−**0.41	**−**0.35	**−**0.02	**−**0.20	0.47	**−**0.41	0.16	0.24	**−**0.23	0.23		**0.63**	**−0.69**	0.27	**−**0.43	0.28	0.15	**−**0.58	0.31
Smois	0.36	0.47	0.31	0.04	**−**0.02	**−**0.43	0.07	**−**0.05	**−**0.27	0.13	0.33	0.02	**−**0.03		**−0.91**	0.66	**−**0.19	**−**0.05	**0.56**	**−0.50**	**0.57**
Sdens	**−**0.32	**−**0.41	**−**0.26	0.03	0.03	0.42	**−**0.10	0.07	0.18	**−**0.21	**−**0.23	**−**0.04	**−**0.02	**−0.95**		**−0.82**	0.37	0.19	**−**0.70	**0.56**	**−**0.63
Bark	0.64	0.07	**0.56**	**−**0.16	0.10	**−**0.21	**−**0.13	**−**0.09	**−**0.13	0.27	**−**0.18	0.31	0.09	**0.53**	**−0.61**		**−**0.24	**−**0.03	**0.86**	**−**0.65	0.64
Slength	0.22	**−**0.05	0.28	0.25	**0.52**	**−**0.21	**−**0.43	0.28	0.03	**−**0.49	0.03	**−**0.08	**−**0.23	**−**0.04	0.13	0.06		0.75	**−**0.03	**−**0.12	0.20
Sdiam	**−**0.06	0.06	**−**0.04	0.05	0.29	**−**0.32	0.00	0.13	0.06	**−**0.20	**−**0.25	0.35	0.10	0.20	**−**0.16	0.11	**0.59**		0.25	**−**0.21	0.41
Vdiam	0.43	0.03	0.39	**−**0.07	0.15	**−**0.44	**−**0.17	**−**0.03	**−**0.33	0.17	**−**0.02	0.16	0.03	**0.52**	**−0.55**	**0.78**	0.24	0.40		**−0.63**	**0.86**
Vdens	**−**0.36	**−**0.34	**−**0.37	**−**0.15	**−**0.07	**0.49**	0.04	0.05	0.08	**−**0.11	**−**0.15	**−**0.08	**−**0.12	**−0.58**	**0.55**	**−**0.46	**−**0.18	**−**0.36	**−**0.63		**−0.51**
Vindex	0.32	0.04	0.29	**−**0.12	0.10	**−**0.38	**−**0.12	0.02	**−**0.43	0.29	**−**0.06	0.14	0.10	**0.55**	**−0.58**	**0.59**	0.33	**0.54**	**0.86**	**−0.57**	

Traits and abbreviations are as in Tables S2 and S3. For all traits, we used *n* = 33 species, except for leaf anatomical and biochemical traits (*n* = 21 species). Only significant (*p* < .05) correlations are shown.

Stems presented higher within‐organ correlations than leaves (Table 1). Pairwise trait correlations between species using PIC values showed that softer stems had high bark mass and moisture, but low vessel density. Softer stems were correlated with large leaves through bark mass, vessel density, and diameter. In addition, softer stems were correlated to leaves with high protein and sucrose contents that were richer in spongy parenchyma, as indicated by the correlations between stem density, moisture, bark, vessel density, vessel diameter, soluble proteins, sucrose, and palisade to spongy ratio. Softer stems also had a high vulnerability index, which was correlated to the cuticle layer. Deciduousness predominated among large‐leaved species, which ranged from 42.85 to 99.14 cm² (Table S2). Despite this, all 21 species showed hypostomatic leaves with reduced spongy intercellular spaces in the dorsiventral mesophyll. Temporal variability of litterfall production was negatively correlated to vessel diameter and positively correlated to sucrose.

**Figure 1:**
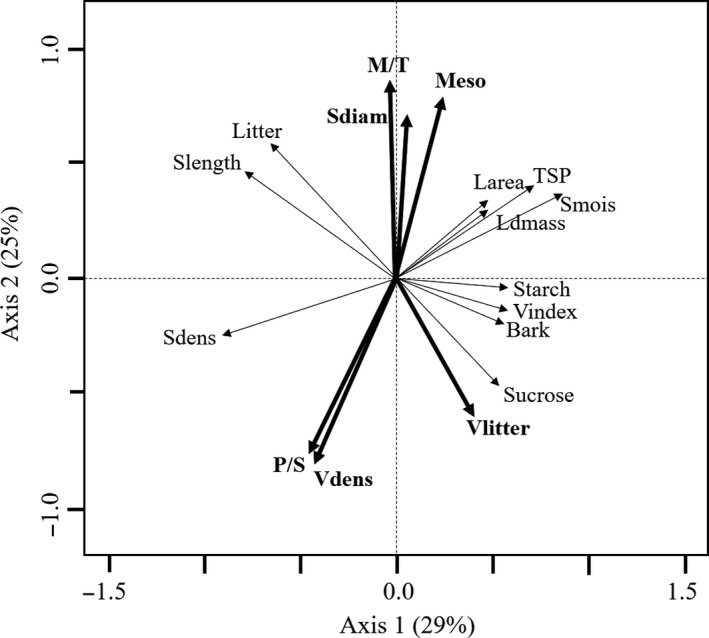
Biplot of functional relationships among leaf and stem traits from multiple factor analysis (MFA) based on phylogenetically independent contrasts (PICs). Traits and abbreviations: Leaf dry mass (Ldmass), leaf area (Larea), litterfall production (Litter), temporal variability in litterfall production (Vlitter), mesophyll layer (Meso), palisade to spongy parenchyma ratio (P/S), mesophyll to total leaf thickness (M/T), starch, sucrose, total soluble protein (TSP),stem moisture (Smois), bark (Bark), stem density (Sdens), vessel density (Vdens), vulnerability index (Vindex), stem length (Slength), and stem diameter (Sdiam). Thin arrows are correlated to axis 1, while thick arrows are correlated to axis 2

Multiple factor analysis produced two independent axes (eigenvalues >1) using 21 species. The axes accounted for 41% of trait variance using the raw dataset and 53% using phylogenetically independent contrasts (Table 2), where most variables had high loadings on both axes. In accordance with the trait–trait correlations, the first MFA axis showed that short plants with high bark dry mass, moisture, and vulnerability to cavitation invested in large leaves with higher synthesis of soluble proteins and sucrose, while plants with longer, dense stems invested in small leaves (Figure 1). Most anatomical leaf traits, the temporal variability of litterfall production, vessel density, and the stem diameter varied independently from the first MFA axis. The MFA for a higher number of species (*n* = 33) using 14 traits (anatomical and biochemical traits excluded) produced only one significant axis (Table S4). This axis was similar to the MFA's main axis using 21 species, but most variables had stronger loadings.

**Table 2:** Multiple factor analysis (MFA) based on raw data and phylogenetically independent contrasts (PICs) for sets of leaf and stem traits of 21 *Restinga* species

	Raw data		PIC values	
	MFA1	MFA2	MFA1	MFA2
Eigenvalue	1.33	1.04	1.53	1.33
% of var.	23	18	29	25
Leaf				
Ldmass	**0.63**	0.23	**0.55**	0.30
Lmois	0.30	**−**0.12	0.21	0.31
Larea	**0.62**	0.50	**0.54**	0.35
SLA	0.10	**0.64**	0.08	0.42
Litter	**−**0.41	0.23	**−0.67**	0.58
Vlitter	**−**0.13	0.16	0.41	**−0.61**
Meso	**−**0.21	**−0.85**	0.25	**0.79**
P/S	0.23	**0.73**	**−**0.42	**−0.82**
M/T	**−**0.48	**−**0.32	**−**0.04	**0.84**
Cuticle	0.32	**−**0.55	0.12	0.23
Starch	0.11	0.16	**0.60**	**−**0.06
Sucrose	0.26	**−**0.2	**0.52**	**−**0.46
TSP	0.02	**−0.66**	**0.72**	0.39
Stem				
Smois	**0.73**	**−**0.20	**0.85**	0.36
Sdens	**−0.76**	0.32	**−0.92**	**−**0.26
Bark	**0.61**	**−**0.07	**0.57**	**−**0.20
Vdiam	**0.83**	**−**0.02	0.44	**−**0.03
Vdens	**−0.78**	0.14	**−**0.46	**−0.77**
Vindex	**0.89**	**−**0.12	**0.60**	**−**0.15
Slength	**−**0.31	**0.67**	**−0.81**	0.46
Sdiam	0.32	0.00	0.05	**0.71**

Significant eigenvalues and percentage of variance explained (axis features [% of var.]) from the MFA axes are shown. High loadings of traits on the MFA axes are shown in boldface. Traits and abbreviations are as in Tables S2 and S3.

**Figure 2:**
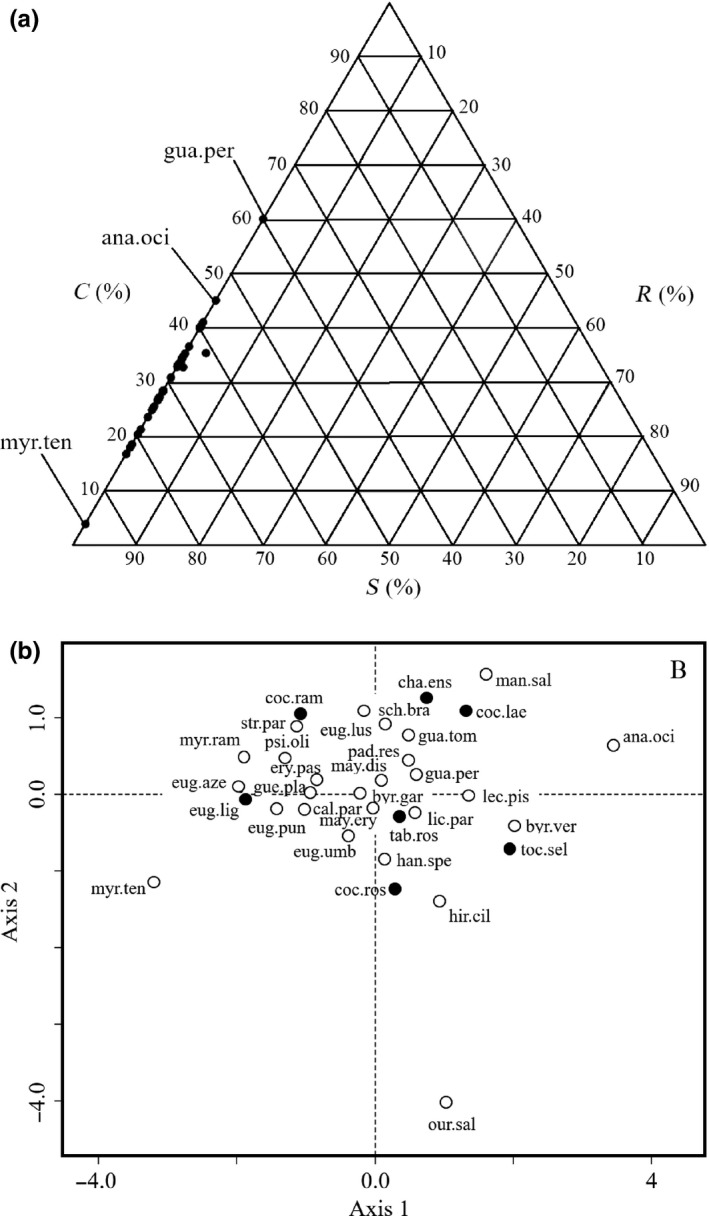
Two representations of the functional space occupied by species of the *Restinga* heath ecosystem in CSR ternary and multiple factor analysis (MFA) plots. The ternary plot (a) is characterized by the leaf area (C‐strategy), leaf dry mass (S‐strategy), and specific leaf area (R‐strategy) according to the CSR classification, while the MFA plot (b) is characterized by multiple covariation of 14 traits. In B, white dots represent evergreen species, while black dots represent semideciduous or deciduous species. Species’ acronyms are as in Table S1

The species were concentrated at the stress‐tolerant end of the competitor‐stress‐tolerant side of the Grime's CSR triangle (Figure 2a and Table S2). The species’ positions on the CSR ternary plot corresponded to the main functional dimension created by the MFA (Figure 2b).

## 4 DISCUSSION

A single axis of trait coordination has been shown to explain from 25% to 60% of the whole‐plant functioning in local communities, landscapes, and even globally (Diaz et al., 2016; Li et al., 2015; Méndez‐Alonzo et al., 2016). We found that leaf traits showed substantial covariation even though their relationships with stem traits were not so strong. Variation of plant form and function was split into two weak and mostly independent economic spectra, as suggested by the small explained variation and similar eigenvalues of the multiple factor analysis’ axes (Dwyer & Laughlin, 2017b). This result places the *Restinga* vegetation among the communities with the lowest whole‐plant trait coordination (Diaz et al., 2016; Li et al., 2015; Méndez‐Alonzo et al., 2016). In addition, results contradicted our expectation that harsh conditions in the coastal sandy plains of northeastern South America would strengthen the degree of within‐community trait coordination by decreasing the viable niche space (Dwyer & Laughlin, 2017a,b; Westoby & Wright, 2006).

Trait independence seems to allow species to better optimize their survival and growth by investing differently in their form and function than strong coordination of traits would (Baraloto et al., 2010; Fortunel et al., 2012). The low trait coordination observed among *Restinga* species might reflect the fact that climate conditions in coastal sandy plains in northeastern South America are not as harsh as other environments in which trait coordination has been tested. Dwyer and Laughlin (2017a) tested the strength of trait coordination in herbaceous communities along a gradient of environmental harshness from savannas to deserts in Western Australia, which presented more severe abiotic conditions than the coastal plain we studied. Although *Restinga* vegetation faces high soil salinity and acidity and poor nutrition, the annual precipitation in the study region averages 1746 mm, and it may act as an important driver of leaf and stem independence. South American coastal plains have been colonized by species from diverse neighboring vegetation types, such as the semi‐arid Caatinga, the Cerrado savanna, and the Amazon and Atlantic rainforests (Scarano, 2002). Species groups with distinct biogeographic origins might show a higher trait diversity to deal with harsh conditions than a community assembled with phylogenetically related species, such as Australian savannas and deserts. Lack of root traits for this functional axis could also explain part of the trait independence seen in our data, as leaf, stem, and root traits are jointly the basis for species strategies (Fortunel et al., 2012). However, homogeneity of soil nutrients distribution in the study area (Silva et al., 2015) makes strong root trait coordination unlikely.

Despite weak trait coordination, the pattern of trait correlations on the MFA's main axis fits well with the global plant economic spectrum, which supports small‐leaved species with dense stems facilitating slower strategies and opposes large‐leaved species with soft stems facilitating faster ecological strategies (Diaz et al., 2016; Reich, 2014). This axis corresponded to the competitor‐stress‐tolerant side of Grime's (1977) CSR triangle. Woody shrubs and trees are often positioned between the C‐S extremities in the CSR triangle, but the stronger S orientation we found for *Restinga* species implies that dune fields and sandy plains greatly favor tolerance to poor soil conditions (low nutrients, high salinity, and low water retention) coupled with high radiation exposure and long dry seasons (Grime, 1997; Pierce et al., 2013). This result also implies that *Restinga* is likely to lack species categorized near the competitor end of the CSR triangle, such as species from mesic vegetation types (i.e., Amazon and Atlantic rainforests), but present more drought‐prone semi‐arid species from the Caatinga and Cerrado savannas.

Some degree of trait coordination characterizing a stress‐tolerant strategy is likely an ecophysiological requirement for plants to colonize *Restinga* heath vegetation and deal with low soil nutrients and marked drought seasonality. After accounting for phylogenetic relatedness, we found that leaf area was negatively correlated to vessel density, while positively correlated to vessel diameter, and bark dry mass; and that stem vulnerability index was correlated to the cuticle layer of leaves. Furthermore, leaf area was negatively correlated to stem density in the multiple factor analysis. These trait relationships reflect trade‐offs related to water movement from stems to leaves (Chave et al., 2009; Méndez‐Alonzo et al., 2016). Regulation of water movement between plant organs is a key functional requirement for plants to maintain hydraulic safety in seasonally dry vegetation, where they have to deal with long periods of water shortage combined with high radiation and heat loads (Markesteijn et al., 2007, 2011; Méndez‐Alonzo et al., 2016; Pivovaroff, Sack, & Santiago, 2014). Hydraulic safety requirements would explain these relationships as well as the prevalence of species with small thick leaves and dense stems in the study region, which is consistent with previous findings in the literature (Jacobsen, Pratt, Ewers, & Davis, 2007; Pivovaroff et al., 2014). Small leaves dissipate more heat and, consequently, have lower transpiration costs than large leaves (Wright et al., 2006). Additionally, dense stems have lower hydraulic efficiency and higher resistance to drought‐induced embolism by air seeding than softer stems (Chave et al., 2009; Scholz et al., 2013).

According to the leaf economic spectrum, higher leaf area is related to cheap resource‐acquisitive leaves that are more efficient in carbon uptake and have lower water economy (Wright et al., 2004). Acquisitive leaves are characterized by high photosynthetic and transpiration rates, low carbon investment, high C:N ratios, and high nutrient turnover (Ishida et al., 2008; Reich, 2014). High concentrations of Rubisco enzyme (reflected in leaf N), biosynthetic precursors (reflected in leaf P), and cellular fuels as sucrose and starch (reflected in leaf C) are also characteristics of acquisitive leaves (Reich et al., 2003). In turn, the high transpiration costs required by large leaves to maintain leaf cooling might decrease their lifespans and induce leaf‐shedding behavior at the first water shortage event (Méndez‐Alonzo et al., 2016; Reich et al., 2003). Although leaf lifespan has been shown to correlate with some phenological traits, it is still debated whether leaf lifespan or other leaf traits are correlated with leaf litterfall production (Li, Liu, & Bao, 2016). However, the correlation between SLA and deciduousness (Méndez‐Alonzo et al., 2016), as well as between SLA and the ecosystem productivity (Reich, 2014), has suggested that litterfall production might also be correlated with the leaf economic spectrum. Acquisitive leaves also investment more in productivity tissues (e.g., palisade parenchyma) than in protective tissues (e.g., epidermis), with the palisade layer containing a greater portion of leaf nitrogen (Li et al., 2015; Markesteijn et al., 2007; Somavilla et al., 2014). Our results partially corroborate these expectations. We found that large leaves (1) had higher synthesis of biosynthetic precursors and cellular fuels (indicative of high photosynthetic activity; Reich et al., 2003) and (2) were more often deciduous than small leaves (indicative of short leaf lifespan; Fu et al., 2012; Méndez‐Alonzo et al., 2016). Large‐leaved species showed less litterfall mass than small‐leaved species, although the strength of this spectrum was weak within the *Restinga* community. However, (3) large leaves were not richer in acquisitive anatomical traits, as suggested by the MFA analysis. Further investigations in systems under distinct degrees of stress will contribute to understand the generality of whole‐plant trait coordination within local communities in stressful environments.

## DATA ACCESSIBILITY

All mean trait data used in this manuscript are present in Tables S1 and S2.

## CONFLICT OF INTEREST

None declared.

## AUTHORS’ CONTRIBUTIONS

JS and AS conceived the ideas, the methodology, and wrote the manuscript; JS collected and analyzed leaf and stem trait data; AC collected litterfall data; EV contributed with the leaf biochemistry analysis; JL contributed with the leaf anatomy analysis. All authors contributed critically to the drafts and gave final approval for publication.

## References

[ece33547-bib-0001] Alvares, C. A. , Stape, J. L. , Sentelhas, P. C. , De Moraes Gonçalves, J. L. , & Sparovek, G. (2013). Koppen's climate classification map for Brazil. Meteorologische Zeitschrift, 22, 711–728. https://doi.org/10.1127/0941-2948/2013/0507

[ece33547-bib-0002] Baraloto, C. , Paine, C. E. T. , Poorter, L. , Beauchene, J. , Bonal, D. , Domenach, A. M. , … Chave, J. (2010). Decoupled leaf and stem economics in rain forest trees. Ecology Letters, 13, 1338–1347. https://doi.org/10.1111/j.1461-0248.2010.01517.x 20807232 10.1111/j.1461-0248.2010.01517.x

[ece33547-bib-0003] Bradford, M. M. (1976). A rapid and sensitive method for the quantitation of microgram quantities of protein utilizing the principle of protein‐dye binding. Analytical Biochemistry, 72, 248–254. https://doi.org/10.1016/0003-2697(76)90527-3 942051 10.1016/0003-2697(76)90527-3

[ece33547-bib-0004] Brancalion, P. H. S. , Vidal, E. , Lavorenti, N. A. , Batista, J. L. F. , & Rodrigues, R. R. (2012). Soil‐mediated effects on potential *Euterpe edulis* (Arecaceae) fruit and palm heart sustainable management in the Brazilian Atlantic Forest. Forest Ecology and Management, 284, 78–85. https://doi.org/10.1016/j.foreco.2012.07.028

[ece33547-bib-0005] Chave, J. , Coomes, D. , Jansen, S. , Lewis, S. L. , Swenson, N. G. , & Zanne, A. E. (2009). Towards a worldwide wood economics spectrum. Ecology Letters, 12, 351–366. https://doi.org/10.1111/ele.2009.12.issue-4 19243406 10.1111/j.1461-0248.2009.01285.x

[ece33547-bib-0006] Clark, D. A. , Brown, S. , Kicklighter, D. W. , Chambers, J. Q. , Thomlinson, J. R. , & Ni, J. (2001). Measuring net primary production in forests: Concepts and field methods. Ecological Applications, 11, 356–370. https://doi.org/10.1890/1051-0761(2001)011[0356:MNPPIF]2.0.CO;2

[ece33547-bib-0007] Diaz, S. , Kattge, J. , Cornelissen, J. H. , Wright, I. J. , Lavorel, S. , Dray, S. , … Gorne, L. D. (2016). The global spectrum of plant form and function. Nature, 529, 167–171. https://doi.org/10.1038/nature16489 26700811 10.1038/nature16489

[ece33547-bib-0008] Dray, S. , & Josse, J. (2015). Principal component analysis with missing values: A comparative survey of methods. Plant Ecology, 216, 657–667. https://doi.org/10.1007/s11258-014-0406-z

[ece33547-bib-0009] Dwyer, J. M. , & Laughlin, D. C. (2017a). Constraints on trait combinations explain climatic drivers of biodiversity: The importance of trait covariance in community assembly. Ecology Letters, 20, 872–882. https://doi.org/10.1111/ele.2017.20.issue-7 28510261 10.1111/ele.12781

[ece33547-bib-0010] Dwyer, J. M. , & Laughlin, D. C. (2017b). Selection on trait combinations along environmental gradients. Journal of Vegetation Science, 28, 672–673. https://doi.org/10.1111/jvs.2017.28.issue-4

[ece33547-bib-0011] Felsenstein, J. (1985). Phylogenies and the comparative method. The American Naturalist, 1, 1–15. https://doi.org/10.1086/284325 10.1086/703055 31094602

[ece33547-bib-0012] Fortunel, C. , Fine, P. V. A. , & Baraloto, C. (2012). Leaf, stem and root tissue strategies across 758 Neotropical tree species. Functional Ecology, 26, 1153–1161. https://doi.org/10.1111/j.1365-2435.2012.02020.x

[ece33547-bib-0013] Fu, P. L. , Jiang, Y. J. , Wang, A. Y. , Brodribb, T. J. , Zhang, J. L. , Zhu, S. D. , & Cao, K. F. (2012). Stem hydraulic traits and leaf water‐stress tolerance are co‐ordinated with the leaf phenology of angiosperm trees in an Asian tropical dry karst forest. Annals of Botany, 110, 189–199. https://doi.org/10.1093/aob/mcs092 22585930 10.1093/aob/mcs092 PMC3380589

[ece33547-bib-0014] Grime, J. P. (1997). Evidence for the existence of three primary strategies in plants and its relevance to ecological and evolutionary theory. The American Naturalist, 111, 1169–1194.

[ece33547-bib-0600] INMET (2014). Instituto Nacional de Meteorologia. Available in http://www.inmet.gov.br/portal/ . Accessed on October 2016.

[ece33547-bib-0015] Ishida, A. , Nakano, T. , Yazaki, K. , Matsuki, S. , Koike, N. , Lauenstein, D. L. , … Yamashita, N. (2008). Coordination between leaf and stem traits related to leaf carbon gain and hydraulics across 32 drought‐tolerant angiosperms. Oecologia, 156, 193–202. https://doi.org/10.1007/s00442-008-0965-6 18297313 10.1007/s00442-008-0965-6

[ece33547-bib-0016] Jacobsen, A. L. , Pratt, R. B. , Ewers, F. W. , & Davis, S. D. (2007). Cavitation resistance among 26 chaparral species of Southern California. Ecological Monographs, 77, 99–115. https://doi.org/10.1890/05-1879

[ece33547-bib-0017] Kleyer, M. , & Minden, V. (2015). Why functional ecology should consider all plant organs: An allocation‐based perspective. Basic and Applied Ecology, 16, 1–9. https://doi.org/10.1016/j.baae.2014.11.002

[ece33547-bib-0018] Lê, S. , Josse, J. , & Husson, F. (2008). FactoMineR: An R package for multivariate analysis. Journal of Statistical Software, 25, 1–18.

[ece33547-bib-0019] Li, F. L. , Liu, X. , & Bao, W. (2016). Leaf lifespan is positively correlated with periods of leaf production and reproduction in 49 herb and shrub species. Ecology and Evolution, 6, 3822–3831. 27398191 10.1002/ece3.2147 PMC4933094

[ece33547-bib-0020] Li, L. , McCormack, M. L. , Ma, C. , Kong, D. , Zhang, Q. , Chen, X. , … Guo, D. (2015). Leaf economics and hydraulic traits are decoupled in five species‐rich tropical‐subtropical forests. Ecology Letters, 18, 899–906. https://doi.org/10.1111/ele.2015.18.issue-9 26108338 10.1111/ele.12466

[ece33547-bib-0021] Markesteijn, L. , Poorter, L. , & Bongers, F. (2007). Light‐dependent leaf trait variation in 43 tropical dry forest tree species. American Journal of Botany, 94, 515–525. https://doi.org/10.3732/ajb.94.4.515 21636421 10.3732/ajb.94.4.515

[ece33547-bib-0022] Markesteijn, L. , Poorter, L. , Bongers, F. , Paz, H. , & Sack, L. (2011). Hydraulics and life history of tropical dry forest tree species : Coordination of species drought and shade tolerance. New Phytologist, 191, 480–495. https://doi.org/10.1111/j.1469-8137.2011.03708.x 21477008 10.1111/j.1469-8137.2011.03708.x

[ece33547-bib-0023] McCready, R. M. , Guggolz, J. , Siliviera, V. , & Owens, H. S. (1950). Determination of starch and amylose in vegetables. Analytical Chemistry, 22, 1156–1158. https://doi.org/10.1021/ac60045a016

[ece33547-bib-0024] Méndez‐Alonzo, R. , Paz, H. , Cruz, R. , Rosell, J. A. , & Olson, M. E. (2016). Coordinated evolution of leaf and stem economics in tropical dry forest trees. Ecology, 93, 2397–2406. 10.1890/11-1213.1 23236911

[ece33547-bib-0025] Muehe, D. , Lima, C. F. , & Lins‐de‐Barros, F. M. (2006) Erosão e propagação do litoral Brasileiro. Brasília, Brazil: Ministério do Meio Ambiente.

[ece33547-bib-0026] Pages, J. (2004). Multiple factor analysis: Main features and application to sensory data. Revista Colombiana de Estadistica, 27, 1–26.

[ece33547-bib-0027] Paradis, E. , Claude, J. , & Strimmer, K. (2004). APE: Analyses of phylogenetics and evolution in R language. Bioinformatics, 20, 289–290. https://doi.org/10.1093/bioinformatics/btg412 14734327 10.1093/bioinformatics/btg412

[ece33547-bib-0028] Pérez‐Harguindeguy, N. , Diaz, S. , Garnier, E. , Lavorel, S. , Poorter, H. , Jaureguiberry, P. , … Cornelissen, J. H. C. (2013). New Handbook for standardized measurement of plant functional traits worldwide. Australian Journal of Botany, 61, 167–234. https://doi.org/10.1071/BT12225

[ece33547-bib-0029] Pierce, S. , Brusa, G. , Vagge, I. , & Cerabolini, B. E. L. (2013). Allocating CSR plant functional types: The use of leaf economics and size traits to classify woody and herbaceous vascular plants. Functional Ecology, 27, 1002–1010. https://doi.org/10.1111/1365-2435.12095

[ece33547-bib-0030] Pierce, S. , Negreiros, D. , Cerabolini, B. E. L. , Kattge, J. , Díaz, S. , Kleyer, M. , … Tampucci, D. (2016). A global method for calculating plant CSR ecological strategies applied across biomes world‐wide. Functional Ecology, 31, 444–457.

[ece33547-bib-0031] Pires, L. A. , De Britez, R. M. , Martel, G. , & Pagano, S. N. (2006). Produção, acúmulo e decomposição da serapilheira em uma restinga da Ilha do Mel, Paranaguá, PR, Brasil. Acta Botanica Brasilica, 20, 173–184. https://doi.org/10.1590/S0102-33062006000100016

[ece33547-bib-0032] Pivovaroff, A. L. , Sack, L. , & Santiago, L. S. (2014). Coordination of stem and leaf hydraulic conductance in southern California shrubs: A test of the hydraulic segmentation hypothesis. New Phytologist, 203, 842–850. https://doi.org/10.1111/nph.12850 24860955 10.1111/nph.12850

[ece33547-bib-0033] R Core Team . (2013). R: A language and environment for statistical computing. Vienna, Austria: R Foundation for Statistical Computing.

[ece33547-bib-0034] Reich, P. B. (2014). The world‐wide “fast‐slow” plant economics spectrum: A traits manifesto. Journal of Ecology, 102, 275–301. https://doi.org/10.1111/1365-2745.12211

[ece33547-bib-0035] Reich, P. B. , Wright, I. J. , Cavender‐bares, J. , Craine, J. M. , Oleksyn, J. , Westoby, M. , … Walters, M. B. (2003). The evolution of plant functional variation: Traits, spectra, and strategies. International Journal of Plant Sciences, 1643, 143–164. https://doi.org/10.1086/374368

[ece33547-bib-0036] Scarano, F. R. (2002). Structure, function and floristic relationships of plant communities in stressful habitats marginal to the Brazilian atlantic rainforest. Annals of Botany, 90, 517–524. https://doi.org/10.1093/aob/mcf189 12324276 10.1093/aob/mcf189 PMC4240375

[ece33547-bib-0037] Scholz, A. , Klepsch, M. , Karimi, Z. , & Jansen, S. (2013). How to quantify conduits in wood? Frontiers in Plant Science, 4, 56. 23507674 10.3389/fpls.2013.00056 PMC3600434

[ece33547-bib-0038] Silva, A. C. , Silva, J. L. A. , & Souza, A. F. (2016). Determinants of variation in heath vegetation structure on coastal dune fields in northeastern South America. Revista Brasileira de Botanica, 39, 605–612.

[ece33547-bib-0039] Silva, J. L. A. , Souza, A. F. , Jardim, J. G. , & Goto, B. T. (2015). Community assembly in harsh environments: The prevalence of ecological drift in the heath vegetation of South America. Ecosphere, 6, 18.

[ece33547-bib-0040] Somavilla, N. S. , Kolb, R. M. , & Rossatto, D. R. (2014). Leaf anatomical traits corroborate the leaf economic spectrum: A case study with deciduous forest tree species. Revista Brasileira de Botanica, 37, 69–82.

[ece33547-bib-0041] Van Buuren, S. , & Groothuis‐Oudshoorn, K. (2011). Multivariate imputation by chained equations. Journal of Statistical Software, 45, 1–67.

[ece33547-bib-0042] Van Handel, E. (1968). Direct microdetermination of sucrose. Analytical Biochemistry, 22, 280–283. https://doi.org/10.1016/0003-2697(68)90317-5 5641848 10.1016/0003-2697(68)90317-5

[ece33547-bib-0043] Vinya, R. , Malhi, Y. , Brown, N. , & Fisher, J. B. (2012). Functional coordination between branch hydraulic properties and leaf functional traits in Miombo woodlands: Implications for water stress management and species habitat preference. Acta Physiologiae Plantarum, 34, 1701–1710. https://doi.org/10.1007/s11738-012-0965-3

[ece33547-bib-0044] Waite, T. A. , & Campbell, L. G. (2006). Controlling the false discovery rate and increasing statistical power in ecological studies. Ecoscience, 13, 439–442. https://doi.org/10.2980/1195-6860(2006)13[439:CTFDRA]2.0.CO;2

[ece33547-bib-0045] Westoby, M. , Falster, D. S. , Moles, A. T. , Vesk, P. A. , & Wright, I. J. (2002). Plant ecological strategies: Some leading dimensions of variation between species. Annual Review of Ecology and Systematics, 33, 125–159. https://doi.org/10.1146/annurev.ecolsys.33.010802.150452

[ece33547-bib-0046] Westoby, M. , & Wright, I. J. (2006). Land‐plant ecology on the basis of functional traits. Trends in Ecology and Evolution, 21, 261–268. https://doi.org/10.1016/j.tree.2006.02.004 16697912 10.1016/j.tree.2006.02.004

[ece33547-bib-0047] Wikstrom, N. , Savolainen, V. , & Chase, M. W. (2001). Evolution of angiosperms: Calibrating the family tree. Proceedings of the Royal Society B, 268, 2211–2220. https://doi.org/10.1098/rspb.2001.1782 11674868 10.1098/rspb.2001.1782 PMC1088868

[ece33547-bib-0048] Wright, I. J. , Falster, D. S. , Pickup, M. , & Westoby, M. (2006). Cross‐species patterns in the coordination between leaf and stem traits, and their implications for plant hydraulics. Physiologia Plantarum, 127, 445–456. https://doi.org/10.1111/j.1399-3054.2006.00699.x

[ece33547-bib-0049] Wright, I. J. , Westoby, M. , Reich, P. B. , Oleksyn, J. , Ackerly, D. D. , Baruch, Z. , … Villar, R. (2004). The worldwide leaf economics spectrum. Nature, 428, 821–827. https://doi.org/10.1038/nature02403 15103368 10.1038/nature02403

