# Supplementary material for: Weak whole‐plant trait coordination in a seasonally dry South American stressful environment
Source: Ecol Evol. 2017 Nov 9;8(1):4–12. doi: 10.1002/ece3.3547 (PMC5756860; doi:10.1002/ece3.3547)
Supplement: Supplementary file 1 [file ECE3-8-4-s001.docx]

# **Supplemental Material**

**Title:** Weak whole-plant trait coordination in a seasonally dry South American stressful environment

**Authors:** José Luiz A. Silva, Alexandre F. Souza, Adriano Caliman, Eduardo Luiz Voigt, and Juliana Espada Lichston

Table S1. Studied species, botanical names and acronyms.

| Family | Species | Code |
| --- | --- | --- |
| Anacardiaceae | *Anacardium occidentale* | ana.oci |
| Malpighiaceae | *Byrsonima gardneriana* | byr.gar |
| Malpighiaceae | *Byrsonima verbascifolia* | byr.ver |
| Fabaceae | *Calliandra parvifolia* | cal.par |
| Fabaceae | *Chamaecrista ensiformis* | cha.ens |
| Polygonaceae | *Coccoloba laevis* | coc.lae |
| Polygonaceae | *Coccoloba ramosissima* | coc.ram |
| Polygonaceae | *Coccoloba rosea* | coc.ros |
| Erythroxylaceae | *Erythroxylum passerinum* | ery.pas |
| Myrtaceae | *Eugenia azeda* | eug.aze |
| Myrtaceae | *Eugenia ligustrina* | eug.lig |
| Myrtaceae | *Eugenia luschnathiana* | eug.lus |
| Myrtaceae | *Eugenia punicifolia* | eug.pun |
| Myrtaceae | *Eugenia umbelliflora* | eug.umb |
| Nyctaginaceae | *Guapira pernambusensis* | gua.per |
| Nyctaginaceae | *Guapira tomentosa* | gua.tom |
| Rubiaceae | *Guettarda platypoda* | gue.pla |
| Apocynaceae | *Hancornia speciosa* | han.spe |
| Chrysobalanaceae | *Hirtella ciliata* | hir.cil |
| Lecythidaceae | *Lecythis pisonis* | lec.pis |
| Chrysobalanaceae | *Licania parvifolia* | lic.par |
| Sapotaceae | *Manilkara salzmannii* | man.sal |
| Celastraceae | *Maytenus distichophylla* | may.dis |
| Celastraceae | *Maytenus erythroxylon* | may.ery |
| Myrtaceae | *Myrcia ramuliflora* | myr.ram |
| Myrtaceae | *Myrciaria tenella* | myr.ten |
| Ochnaceae | *Ouratea salicifolia* | our.sal |
| Sapotaceae | *Padrosia restingae* | pad.res |
| Myrtaceae | *Psidium oligospermum* | psi.oli |
| Schoepfiaceae | *Schoepfia brasiliensis* | sch.bra |
| Loganiaceae | *Strychnos parvifolia* | str.par |
| Bignoniaceae | *Tabebuia roseoalba* | tab.ros |
| Rubiaceae | *Tocoyena sellowiana* | toc.sel |


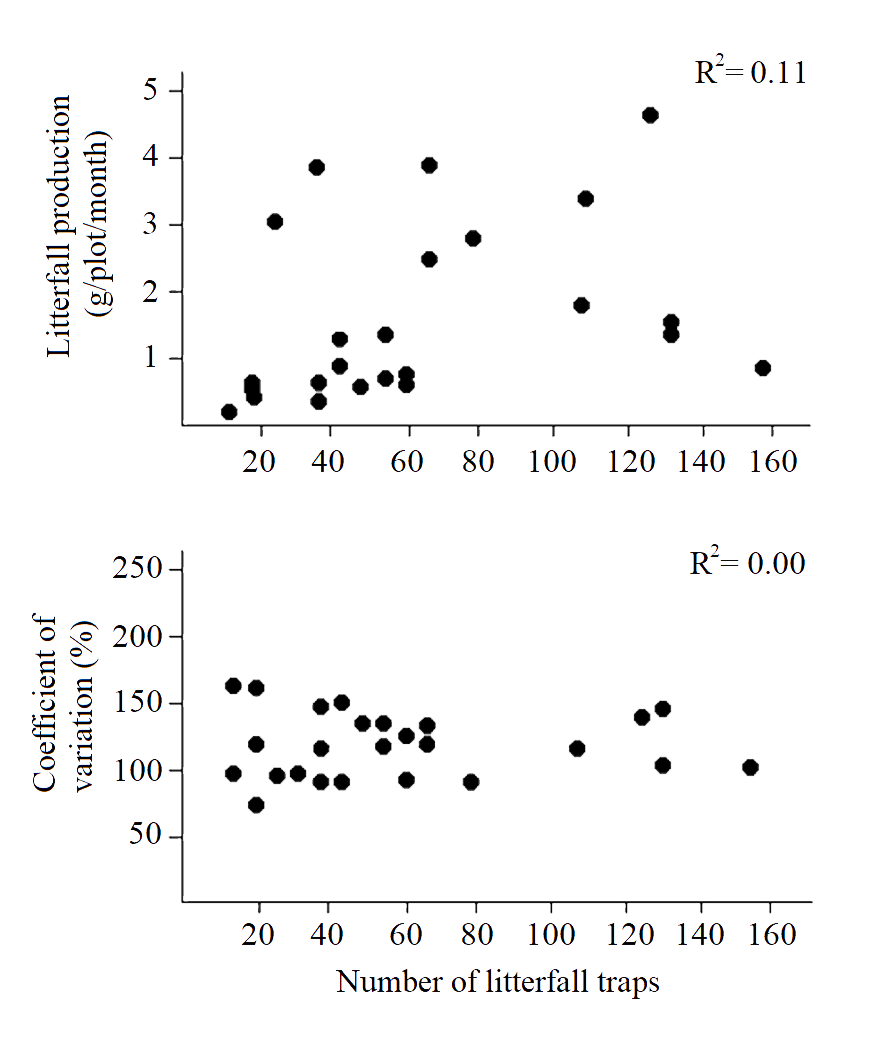


Figure S1. The correlation between the mean and the coefficient of variation of litterfall production, and the number of litterfall traps in which *Restinga* species were found. Species-specific mean litter traits are represented by black dots.


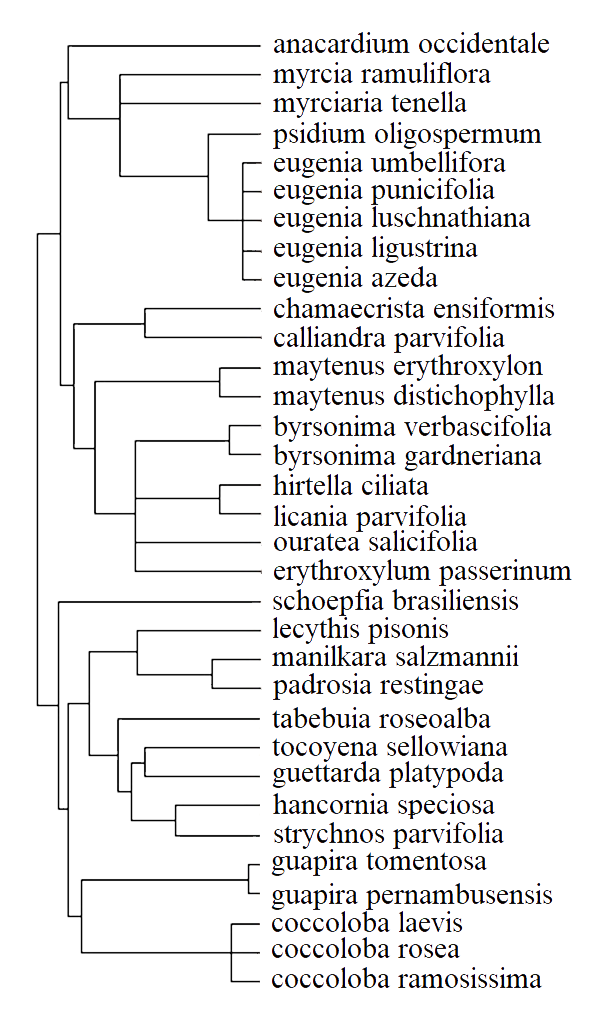


Figure S2. Phylogenetic tree of 33 native woody species from the *Restinga* heath vegetation, Northeastern Brazil.

Table S2. Mean trait values for leaf morphology, litterfall productivity, stem morphology, stem anatomy and the CSR ecological strategies of 33 *Restinga* species at Parnamirim, Northeastern Brazil. The minimum, maximum, as well as the order of magnitude of trait variation are also shown bellow.

|  | Leaf morphology | | | | |  | Litterfall productivity | | |  | Stem morphology | | | | |  | Stem anatomy | | |  | Strategy |
| --- | --- | --- | --- | --- | --- | --- | --- | --- | --- | --- | --- | --- | --- | --- | --- | --- | --- | --- | --- | --- | --- |
| Species | | Ldmass | Larea | SLA | Lmois |  | Litter | Vlitter | Lshed |  | Smois | Bark | Sdens | Slength | Sdiam |  | Vdiam | Vdens | Vindex |  | CSR |
|  | | g | cm² | cm².g^-1^ | % |  | g.ind. month^-1^ | % |  |  | % | % | g.cm^-^³ | m | mm |  | µm | vessel.mm^-^² |  |  |  |
| ana.oci | | 1.08 | 83.07 | 78.33 | 54 |  | - | - | E |  | 0.5 | 0.44 | 0.5 | 7.32 | 88.16 |  | 161.3 | 7.15 | 25.23 |  | SC |
| byr.gar | | 0.1 | 8.23 | 83.14 | 49.87 |  | 1.48 | 98.4 | E |  | 0.38 | 0.26 | 0.68 | 3.43 | 47.23 |  | 98.71 | 33.67 | 2.98 |  | S/SC |
| byr.ver | | 1.16 | 73.63 | 67.37 | 61 |  | - | - | E |  | 0.51 | 0.5 | 0.49 | - | - |  | 79.28 | 30.18 | 2.62 |  | SC |
| cal.par | | 0.13 | 24.1 | 137.8 | 30 |  | 0.51 | 132.2 | E |  | 0.36 | 0.18 | 0.71 | 4.13 | 38.53 |  | 53.66 | 57.19 | 0.97 |  | S/SC |
| cha.ens | | 0.3 | 43.03 | 148.3 | 50.87 |  | 3.91 | 128.7 | D |  | 0.26 | 0.23 | 0.87 | 4.65 | 65.36 |  | 82.7 | 37.42 | 2.3 |  | S/SC |
| coc.lae | | 1.35 | 99.14 | 79.44 | 57 |  | 3.87 | 88.7 | SD |  | 0.34 | 0.36 | 0.74 | 4.62 | 46.65 |  | 84.19 | 50.4 | 1.81 |  | SC |
| coc.ram | | 0.13 | 10.24 | 79.75 | 51.09 |  | 4.64 | 136.0 | D |  | 0.3 | 0.11 | 0.81 | 4.23 | 46.01 |  | 47.65 | 120.5 | 0.4 |  | S/SC |
| coc.ros | | 1.03 | 79.96 | 75.49 | 55 |  | - | 200.0 | SD |  | 0.31 | 0.27 | 0.8 | 3.39 | 42.74 |  | 55 | 50.25 | 1.11 |  | S/SC |
| ery.pas | | 0.12 | 10.3 | 86.76 | 47.63 |  | 0.66 | 113.3 | E |  | 0.33 | 0.25 | 0.75 | 3.85 | 43.82 |  | 54.11 | 121.5 | 0.45 |  | S/SC |
| eug.aze | | 0.08 | 3.49 | 42.37 | 46.43 |  | 2.48 | 116.5 | E |  | 0.3 | 0.1 | 0.81 | 3.03 | 48.26 |  | 40.86 | 110.5 | 0.42 |  | S |
| eug.lig | | 0.08 | 4.52 | 56.54 | 44.77 |  | 1.31 | 133.0 | E |  | 0.24 | 0.18 | 0.86 | 3.11 | 48.97 |  | 41.63 | 101.4 | 0.42 |  | S/SC |
| eug.lus | | 0.12 | 9.8 | 100.6 | 56 |  | 0.84 | 87.6 | E |  | 0.33 | 0.11 | 0.81 | 4.55 | 64.91 |  | 44.92 | 53.25 | 0.94 |  | S/SC |
| eug.pun | | 0.06 | 3.06 | 58.83 | 48 |  | 0.43 | 159.5 | E |  | 0.28 | 0.23 | 0.78 | 4 | 62.6 |  | 40.41 | 138.3 | 0.31 |  | S |
| eug.umb | | 0.16 | 9.58 | 59.44 | 55.62 |  | - | - | E |  | 0.37 | 0.19 | 0.68 | 2.65 | 54.71 |  | 73.52 | 50.19 | 1.52 |  | S/SC |
| gua.per | | 0.11 | 11.33 | 105.8 | 80.76 |  | 0.54 | 70.6 | E |  | 0.51 | 0.06 | 0.51 | 2.97 | 55.4 |  | 61.89 | 19.7 | 3.21 |  | C/SC |
| gua.tom | | 0.3 | 25.74 | 82.17 | 56.57 |  | 3.39 | 113.1 | E |  | 0.32 | 0.09 | 0.74 | 4.2 | 55.08 |  | 46.94 | 10.49 | 4.7 |  | S/SC |
| gue.pla | | 0.17 | 12.18 | 67.69 | 54.52 |  | 1.32 | 141.5 | SD |  | 0.3 | 0.17 | 0.8 | 3.65 | 47.77 |  | 47.62 | 103.1 | 0.49 |  | S/SC |
| han.spe | | 0.08 | 6.5 | 85.68 | 57 |  | 0.58 | 145.7 | E |  | 0.48 | 0.26 | 0.52 | 3.15 | 64.6 |  | 52.93 | 20.7 | 1.93 |  | S/SC |
| hir.cil | | 0.2 | 12.17 | 57.86 | 50.81 |  | 0.33 | 112.7 | E |  | 0.43 | 0.3 | 0.57 | 3.08 | 58 |  | 124.6 | 6.84 | 18.6 |  | S/SC |
| lec.pis | | 0.35 | 30.96 | 91.34 | 55 |  | 0.47 | 116.3 | E |  | 0.31 | 0.42 | 0.76 | 4.39 | 65.6 |  | 119.0 | 14.43 | 8.64 |  | S/SC |
| lic.par | | 0.26 | 17.25 | 67.53 | 42 |  | 0.18 | 95.0 | E |  | 0.3 | 0.21 | 0.84 | 4.16 | 60.43 |  | 115.0 | 20.45 | 6.05 |  | S/SC |
| man.sal | | 0.36 | 23.23 | 64.6 | 52.2 |  | 9.98 | 94.9 | E |  | 0.36 | 0.31 | 0.7 | 5.5 | 78.83 |  | 114.6 | 18.97 | 6.59 |  | S/SC |
| may.dis | | 0.54 | 28.92 | 56.37 | 53.63 |  | - | - | E |  | 0.29 | 0.24 | 0.81 | 3.74 | 38.85 |  | - | - | - |  | S/SC |
| may.ery | | 0.46 | 23.49 | 52.21 | 52.92 |  | 0.81 | 97.6 | E |  | 0.29 | 0.24 | 0.81 | 3.39 | 43 |  | 73.09 | 38.29 | 2.02 |  | S/SC |
| myr.ram | | 0.06 | 3.72 | 58.31 | 43.74 |  | 1.76 | 113.5 | E |  | 0.24 | 0.08 | 0.87 | 3.31 | 52.91 |  | 39.19 | 60.94 | 0.65 |  | S |
| myr.ten | | 0.01 | 0.99 | 83.63 | 44 |  | 0.24 |  | E |  | 0.3 | 0.04 | 0.79 | 2.1 | 58.6 |  | 39.86 | 131.0 | 0.32 |  | S |
| our.sal | | 0.62 | 34.2 | 54.58 | 48.05 |  | - | - | E |  | 0.48 | 0.65 | 0.38 | 1.56 | 50.19 |  | 166.3 | 11.84 | 14.62 |  | S/SC |
| pad.res | | 0.35 | 23.86 | 66.84 | 46.99 |  | 0.68 | 88.0 | E |  | 0.34 | 0.19 | 0.76 | 4.27 | 63.8 |  | 61.83 | 35.29 | 1.82 |  | S/SC |
| psi.oli | | 0.1 | 7.63 | 75.63 | 48 |  | 1.23 | 148.6 | E |  | 0.28 | 0.05 | 0.84 | 3.83 | 57.23 |  | 47.23 | 96.94 | 0.49 |  | S/SC |
| sch.bra | | 0.16 | 11.02 | 69.04 | 60.88 |  | 2.78 | 86.9 | E |  | 0.35 | 0.2 | 0.64 | 3.88 | 56.04 |  | 46.49 | 29.52 | 1.7 |  | S/SC |
| str.par | | 0.05 | 4.99 | 102.1 | 52 |  | 3.03 | 92.6 | E |  | 0.3 | 0.12 | 0.73 | 3.77 | 46.78 |  | 65.08 | 71.59 | 0.95 |  | S/SC |
| tab.ros | | 0.47 | 42.85 | 88.64 | 53.46 |  | 0.59 | 122.8 | D |  | 0.36 | 0.22 | 0.65 | 4.2 | 49.51 |  | 68.51 | 54.16 | 1.29 |  | S/SC |
| toc.sel | | 0.88 | 85.43 | 91.05 | 65 |  | - | - | D |  | 0.37 | 0.25 | 0.69 | - | - |  | 76.06 | 37.08 | 2.13 |  | SC |
|  | |  |  |  |  |  |  |  |  |  |  |  |  |  |  |  |  |  |  |  |  |
| Min | | 0.01 | 0.99 | 42.37 | 30 |  | 0.18 | 70.61 | - |  | 0.24 | 0.04 | 0.38 | 1.56 | 38.53 |  | 39.19 | 6.84 | 0.31 |  | - |
| Max | | 1.35 | 99.14 | 148.3 | 80.76 |  | 9.99 | 200.0 | - |  | 0.51 | 0.65 | 0.87 | 7.32 | 88.16 |  | 166.3 | 138.3 | 25.23 |  | - |
| Magnitude | | 135 | 100.1 | 3.5 | 2.69 |  | 9.81 | 2.84 | - |  | 2.12 | 16.2 | 2.28 | 4.69 | 2.28 |  | 4.24 | 20.22 | 81.38 |  | - |

*Notes:* Traits and abbreviations: Leaf dry mass (Ldmass), leaf area (Larea), specific leaf area (SLA), leaf moisture (Lmois), stem moisture (Smois), bark (Bark), stem density (Sdens), vessel diameter (Vdiam), vessel density (Vdens), vulnerability index (Vindex), stem length (Slength), stem diameter (Sdiam), litter production (Litter), variability in litter production (Vlitter), leaf-shedding behavior (Lshed) [E=evergreen, SD= semi-deciduous, D=deciduous], and the CSR plant strategy according to Pierce *et al.*(2013). Species acronyms are as in Table S1.

Table S3. Mean trait values of leaf anatomy and biochemistry from 21 *Restinga* species. Minimum, maximum and the order of magnitude of trait variation are shown below.

|  | Leaf anatomy | | | |  | Leaf biochemistry | | |
| --- | --- | --- | --- | --- | --- | --- | --- | --- |
|  | Meso | Cut | P/S | M/T |  | Starch | Sucrose | TSP |
| Species | µm | µm | % | % |  | mg.g DM^-1^ | mg.g DM^-1^ | mg.g DM^-1^ |
| byr.gar | 183.93 | 5.68 | 0.65 | 0.72 |  | 34.53 | 14.85 | 12.08 |
| cal.par | - | - | - | - |  | 55.19 | 11.9 | 3.84 |
| cha.ens | 129.93 | 5.64 | 0.66 | 0.72 |  | 26.88 | 25.66 | 6.11 |
| coc.ram | 237.59 | 3.61 | 0.87 | 0.73 |  | 17.82 | 6.54 | 8.81 |
| ery.pas | 208.18 | 4.65 | 0.58 | 0.76 |  | 36.34 | 10.3 | 5.41 |
| eug.aze | 418.76 | 9.61 | 0.17 | 0.89 |  | 21.67 | 26 | 5.84 |
| eug.lig | 376.43 | 9.02 | 0.23 | 0.88 |  | 21.93 | 26.4 | 16.9 |
| eug.umb | 427.17 | 6.74 | 0.49 | 0.92 |  | 13.78 | 12.08 | 9.56 |
| gua.per | 368.53 | 6.26 | 0.45 | 0.59 |  | 60.69 | 7.7 | 6.98 |
| gua.tom | 238.24 | 8.52 | 0.82 | 0.84 |  | 14.67 | 6.81 | 6.03 |
| gue.pla | 118.08 | 5.38 | 2.00 | 0.64 |  | 11.65 | 22.4 | 1.97 |
| han.spe | 285.04 | 5.68 | 0.65 | 0.87 |  | 21.53 | 27.35 | 9.9 |
| hir.cil | 182.47 | 6.84 | 0.89 | 0.57 |  | 32.68 | 22.25 | 10.55 |
| man.sal | 289.21 | 4.62 | 0.75 | 0.81 |  | 13.74 | 17.24 | 7.46 |
| may.dis | 291.09 | - | 0.42 | 0.71 |  | 14.76 | 15 | 9.01 |
| may.ery | 316.23 | 7.3 | 0.4 | 0.72 |  | 33.41 | 15.32 | 10 |
| myr.ram | 298.48 | 2.8 | 0.64 | 0.89 |  | 20.51 | 7.38 | 6.99 |
| our.sal | 285.28 | 9.48 | 0.4 | 0.76 |  | 15.24 | 20.74 | 7.34 |
| pad.res | 178.17 | 6.18 | 0.69 | 0.79 |  | 23.58 | 29.02 | 8.72 |
| sch.bra | 366.86 | 6.49 | 0.67 | 0.88 |  | 20.22 | 20.34 | 14.16 |
| tab.ros | 168 | 4.99 | 0.86 | 0.8 |  | 50.81 | 17.09 | 2.71 |
|  |  |  |  |  |  |  |  |  |
| Min | 118.08 | 2.8 | 0.17 | 0.57 |  | 11.65 | 6.54 | 1.97 |
| Max | 427.17 | 9.61 | 2.00 | 0.92 |  | 60.69 | 29.02 | 16.9 |
| Magnitude | 3.62 | 3.43 | 11.76 | 1.61 |  | 5.21 | 4.44 | 8.57 |

*Notes:* Traits and abbreviations: Mesophyll thickness (Meso), cuticle thickness (Cut), palisade layer per spongy parenchyma layer (P/S), mesophyll layer per total leaf thickness (M/T), starch content (Starch), sucrose content (Sucrose), and total soluble protein (TSP). Species acronyms are as in Table S1.


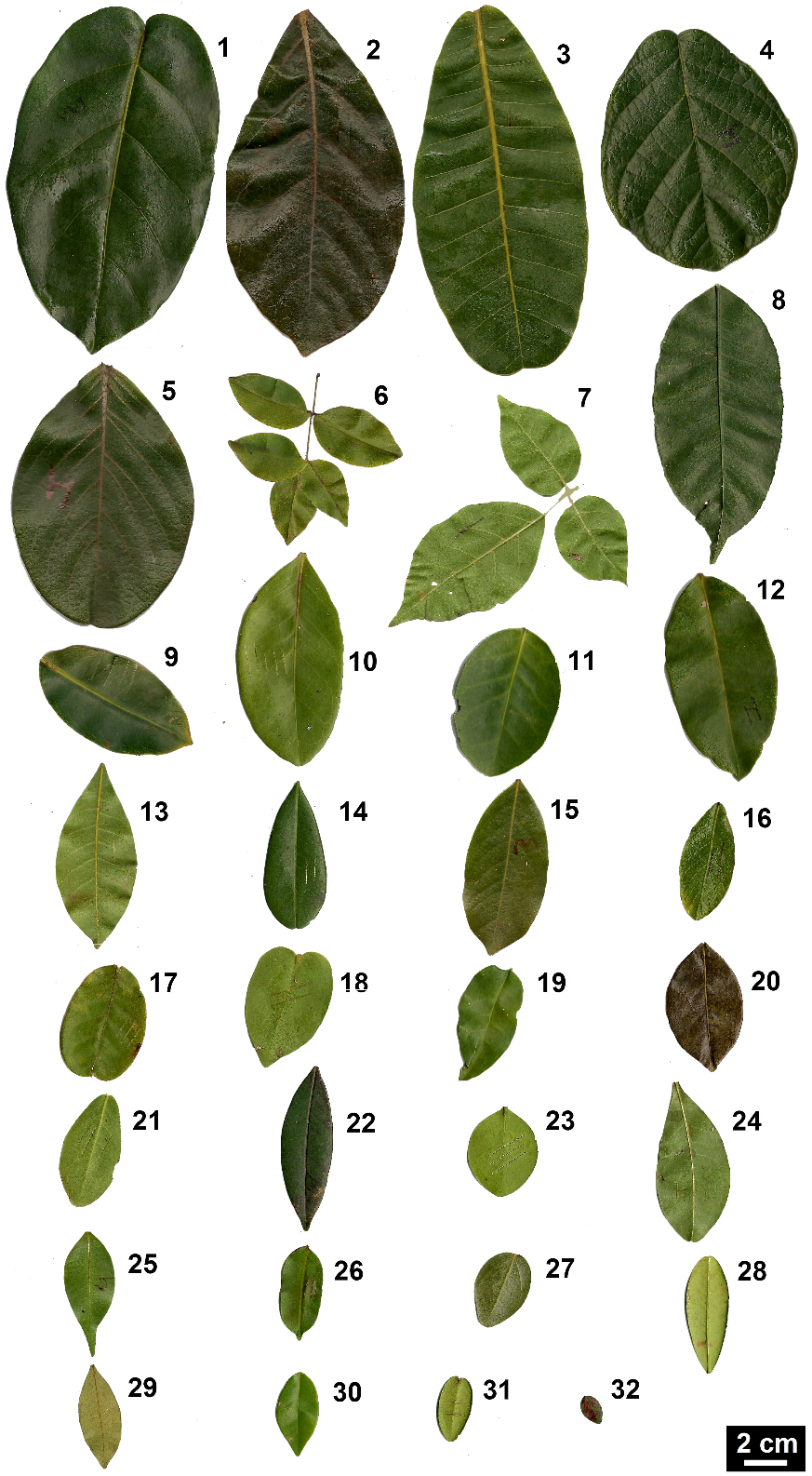


Figure S3. Leaves from the studied species. Species are ordered from the largest to the smallest leaf area: 1-*Coccoloba laevis,* 2-*Tocoyena sellowiana,* 3-*Anacardium occidentale,* 4-*Coccoloba rosea,* 5-*Byrsonima verbascifolia,* 6-*Chamaecrista ensiformis,* 7-*Tabebuia roseoalba,* 8-*Lecythis pisonis,* 9-*Ouratea salicifolia,* 10-*Maytenus distichophylla,* 11-*Maytenus erythroxylon,* 12-*Guapira tomentosa,* 13-*Padrosia restingae,* 14-*Manilkara salzmannii,* 15-*Licania parvifolia,* 16-*Guettarda platypoda,* 17-*Hirtella ciliata,* 18-*Guapira pernambusensis,* 19-*Schoepfia brasiliensis,* 20-*Erythroxylum passerinum,* 21-*Coccoloba ramosissima,* 22-*Eugenia luschnathiana,* 23-*Eugenia umbelliflora,* 24-*Byrsonima gardneriana,* 25-*Psidium oligospermum,* 26-*Hancornia speciosa,* 27-*Strychnosparvifolia,* 28-*Eugenia ligustrina,* 29-*Eugenia punicifolia,* 30-*Myrcia ramuliflora,* 31-*Eugenia azeda, and* 32-*Myrciaria tenella.*


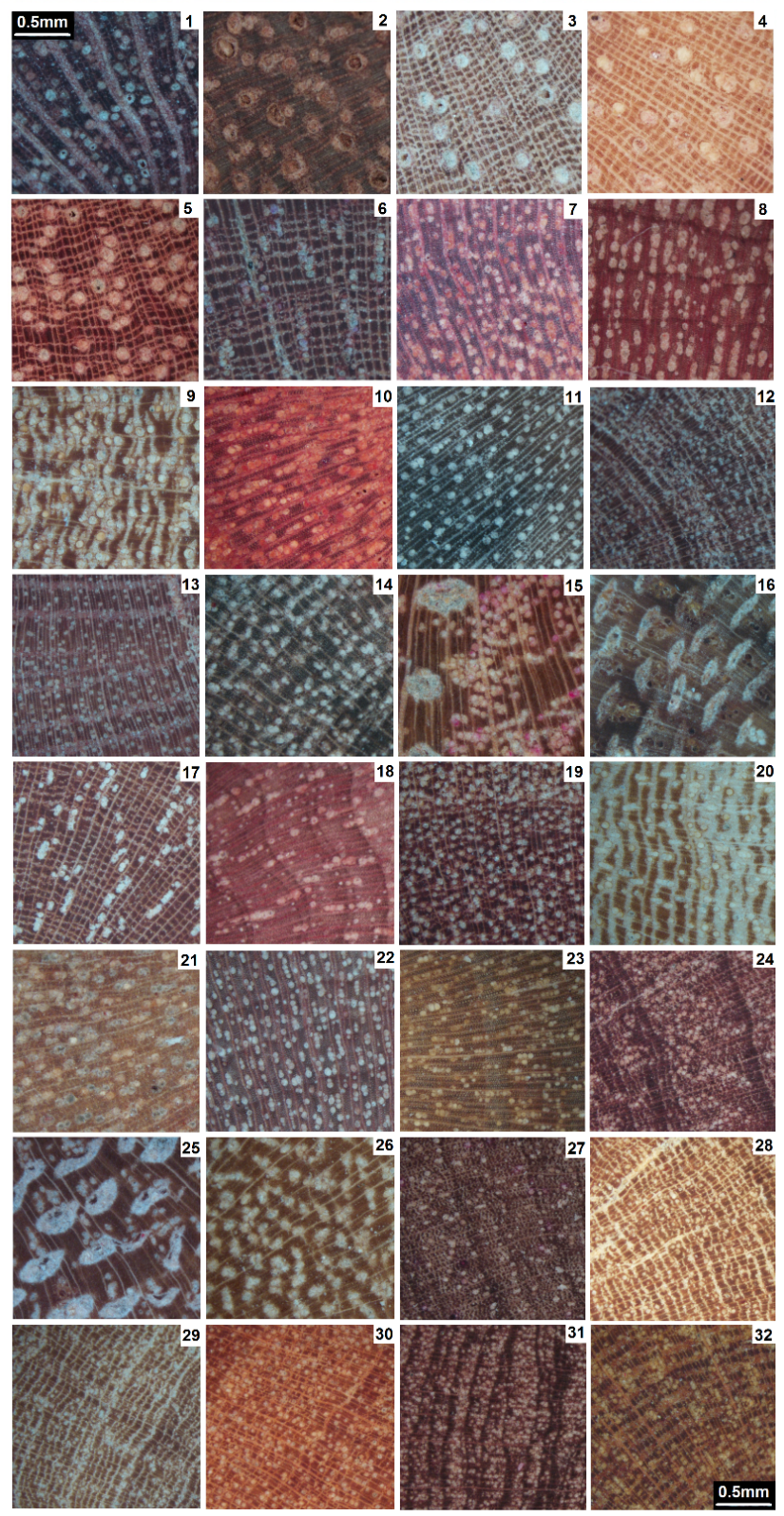


Figure S4. Panel of cross-anatomical stem sections from *Restinga* species. Species are ordered from the widest to the narrowest xylem vessel diameter: 1-*Ouratea salicifolia,* 2-*Anacardium occidentale,* 3-*Hirtella ciliata,* 4-*Lecythis pisonis,* 5-*Licania parvifolia,* 6-*Manilkara salzmannii,* 7-*Byrsonima gardneriana,* 8-*Coccoloba laevis,* 9-*Chamaecrista ensiformis,* 10-*Byrsonima verbascifolia,* 11-*Tocoyena sellowiana,* 12-*Eugenia umbelliflora,* 13-*Maytenus distichophylla/erythroxylon,* 14-*Tabebuia roseoalba,* 15-*Strychnosparvifolia,* 16-*Guapira pernambusensis,* 17-*Padrosia restingae,* 18-*Coccoloba rosea,* 19-*Erythroxylum passerinum,* 20-*Calliandra parvifolia,* 21-*Hancornia speciosa,* 22-*Coccoloba ramosissima,* 23-*Guettarda platypoda,* 24-*Psidium oligospermum,* 25-*Guapira tomentosa,* 26-*Schoepfia brasiliensis,* 27-*Eugenia luschnathiana,* 28-*Eugenia ligustrina,* 29-*Eugenia azeda,* 30-*Eugenia punicifolia,* 31- *Myrciaria tenella, and* 32-*Myrcia ramuliflora*.

**
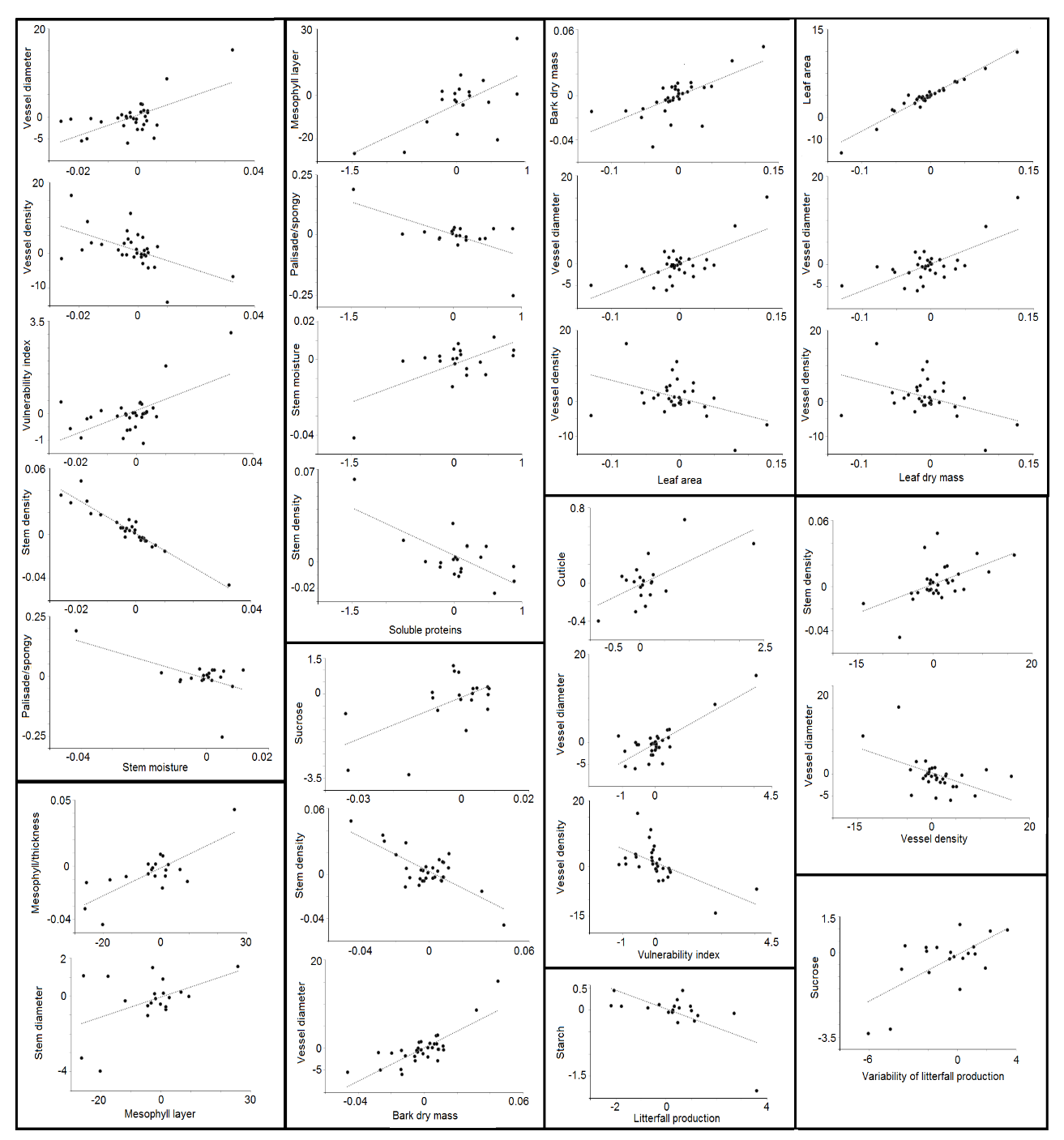
**

Figure S5. Significant pairwise correlations (P ≤ 0.05) between leaf and stem traits using phylogenetic independent contrasts values.

Table S4. Table 2. Multiple Factor Analysis (MFA) based on raw data and phylogenetically independent contrasts (PICs) for sets of leaf and stem traits of 33 *Restinga* species.

|  | MFA 1  (raw data) | MFA 1  (PICs) |
| --- | --- | --- |
| Eigenvalue | 1.80 | 2.26 |
| % of var. | 33 | 43 |
| Leaf |  |  |
| Ldmass | **0.80** | **0.90** |
| Lmois | **0.45** | **0.60** |
| Larea | **0.80** | **0.89** |
| SLA | 0.07 | 0.05 |
| Litter | 0.28 | 0.11 |
| Vlitter | 0.06 | -0.17 |
| Stem |  |  |
| Smois | **0.62** | **0.62** |
| Sdens | **-0.57** | **-0.56** |
| Bark | **0.69** | **0.71** |
| Vdiam | **0.69** | **0.81** |
| Vdens | **-0.67** | **-0.62** |
| Vindex | **0.58** | **0.75** |
| Slength | **0.64** | **0.78** |
| Sdiam | **0.63** | **0.84** |
